# Application of 3D Printing in Preoperative Planning

**DOI:** 10.3390/jcm10050917

**Published:** 2021-02-26

**Authors:** Nicole Segaran, Gia Saini, Joseph L. Mayer, Sailen Naidu, Indravadan Patel, Sadeer Alzubaidi, Rahmi Oklu

**Affiliations:** 1 Minimally Invasive Therapeutics Laboratory, Department of Vascular and Interventional Radiology, Mayo Clinic, Phoenix, AZ 85054, USA; segaran.nicole@mayo.edu (N.S.); saini.gia@mayo.edu (G.S.); 2 3D Innovations Laboratory, Mayo Clinic Arizona, 5711 E. Mayo Blvd. Support Services Building, Phoenix, AZ 85054, USA; mayer.joseph@mayo.edu; 3 Department of Radiology, Mayo Clinic, Phoenix, AZ 85054, USA; naidu.sailen@mayo.edu (S.N.); patel.indravadan@mayo.edu (I.P.); alzubaidi.sadeer@mayo.edu (S.A.)

**Keywords:** 3D printing, preoperative planning, medical imaging, technology, anatomical models

## Abstract

Preoperative planning is critical for success in the surgical suite. Current techniques for surgical planning are limited; clinicians often rely on prior experience and medical imaging to guide the decision-making process. Furthermore, two-dimensional (2D) presentations of anatomical structures may not accurately portray their three-dimensional (3D) complexity, often leaving physicians ill-equipped for the procedure. Although 3D postprocessed images are an improvement on traditional 2D image sets, they are often inadequate for surgical simulation. Medical 3D printing is a rapidly expanding field and could provide an innovative solution to current constraints of preoperative planning. As 3D printing becomes more prevalent in medical settings, it is important that clinicians develop an understanding of the technologies, as well as its uses. Here, we review the fundamentals of 3D printing and key aspects of its workflow. The many applications of 3D printing for preoperative planning are discussed, along with their challenges.

## 1. Introduction

Preoperative planning is important for surgical success; it can help to reduce the risks and time spent in the surgical suite [1]. Prior experience and medical imaging are the cornerstones of standard preoperative planning, but their lack of hands-on preparation and visualization capabilities may leave physicians ill-equipped for surgery. Commonly used modalities for surgical planning such as magnetic resonance imaging (MRI) and computed tomography (CT) are unable to accurately portray the complexity of anatomical structures, leaving physicians to conceptualize two-dimensional (2D) reconstructions into three dimensions. Virtual three-dimensional (3D) renderings have made advancements in the efficacy of preoperative planning, yet often fail to fully replicate the reality of patient anatomy [2]. In addition, their intangibility makes surgical simulation difficult, especially for complex surgeries [3].

An innovative solution for preoperative planning and its current limitations is presented by 3D printing. A rapidly developing technology, 3D printing allows for the layer-by-layer construction of anatomically detailed models [4]. It has a multitude of applications in clinical settings, particularly in the creation of patient-specific models for surgical planning. One systematic review found that 82% of studies on 3D printing and preoperative planning noted better surgical outcomes when 3D-printed models were employed instead of standard preoperative planning; more than 50% of studies in the review demonstrated a decrease in operation duration [3].

Here, we aim to give the reader an understanding of 3D printing and its applications towards preoperative planning. We provide an in-depth explanation of the fundamentals of 3D printing, its process, and its uses, as well as its benefits as reported in the literature. Lastly, we discuss the challenges and limitations of 3D printing, as well as future advances that may further improve this technology for clinical practice.

## 2. Fundamentals of 3D Printing

In order to facilitate the use of 3D printing in clinical settings, it is important for physicians to familiarize themselves with the technologies, materials, and techniques behind this process. The methodology of 3D printing differs with desired outcome, and certain methods are better suited for recreating certain anatomies. Proper selection of technique and materials is required in order to create an accurate model for clinical use. Thus, it is important for physicians to establish a holistic understanding of the technical aspects of 3D printing.

The technique of 3D printing, also referred to as additive manufacturing or rapid prototyping, was originally pioneered in the 1980s for industrial and engineering purposes [1]. As its technologies continue to advance, 3D printing is being increasingly applied to medical settings. It allows for the conversion of 2D medical images into a 3D, printed construct. During the printing process, 3D printers read digital models as “parts” constrained by surfaces and enclosed space. Therefore, medical images must be converted into compatible 3D file formats, for example stereolithography (STL) files. STL files define surfaces as a series of triangles or “facets.” In order to convert images like CT scans and MRIs into the STL format, the displayed anatomies must be correctly segmented by tissue and pathology. Only after this segmentation may a 3D model be created and printed. Therefore, the process of creating 3D-printed medical models can be broken down into three steps:

1. Image acquisition, where medical imaging of the patient is obtained;
2. Image segmentation and postprocessing, where 2D images are converted into a compatible, 3D file;
3. The 3D printing, where this digital 3D model is printed into a physical structure [5].

### 2.1. Image Acquisition

The precision of 3D models depends on the quality of the medical imaging used to create them [2]. High-quality image acquisition is a prerequisite for accurate 3D models. Some technical standards on image quality have been established, such as the 2012 European Society of Urogenital Radiology (ESUR) guidelines on prostate MRI acquisition, which were created due to the emphasized need for high-quality MRI in that setting [6]. In addition, the Radiological Society of North America Special Interest Group on 3D printing (RSNA 3D Printing SIG) has published general guidelines on image acquisition for 3D printing [7]. However, large differences in imaging quality remain between institutions, resulting in models with suboptimal sensitivity and specificity [6].

**Figure 1:**
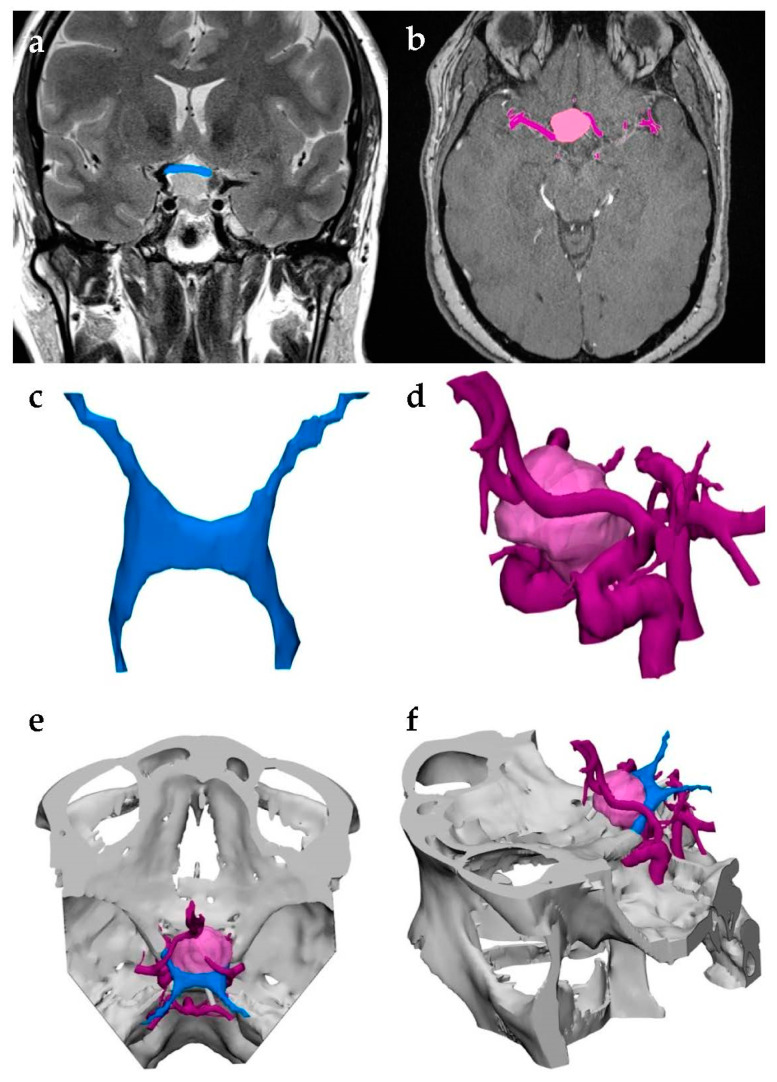
Multimodal image acquisition for three-dimensional (3D) printing. This model was used to visualize a brain tumor and distinguish it from the adjacent optic nerve and surrounding vasculature. (**a**) The optic nerve was captured using magnetic resonance imaging (MRI) and segmented in blue. (**b**) Computed tomography (CT) allowed physicians to identify the neoplasm’s borders, highlighted in pink, along with the neighboring vessels, highlighted in purple. (**c**,**d**) The segmented anatomies were converted to stereolithography (STL) files and (**e**,**f**) merged into a single model to be 3D-printed.

Any volumetric imaging that differentiates between various tissues may be used to generate a 3D model; however, CT is the most common imaging method for models due to its high signal-to-noise ratio (SNR), reasonable soft tissue contrast, and excellent spatial resolution. In some cases, multiple modalities are used to capture different aspects of the anatomy being modeled (Figure 1) [5]. Adjusting the imaging process to increase SNR and spatial resolution is critical and requires specific considerations per modality. Minor adjustments, for instance setting slice thickness lower than collimation in CT scans and thus causing overlap, could greatly improve modeling results [7]. Regardless of the type of imaging, it is important to also consider the purpose of the model being created. Ideally, the volume elements (voxels) in CT data should be isotropic, i.e., the dimensions of the voxel should be identical in three planes. The dimensions in the x- and y-planes of the scan are defined by the detector size in CT whereas the dimension of the z-plane, referred to as “slice thickness”, is more easily adjustable. Optimal isotropic voxel size is critical for generating a quality model. If the voxel size of the acquired images is too thick, the model will be inaccurate; if it is too thin, the model will require unnecessary patient exposure to radiation, as well as considerable segmentation and postprocessing time. Generally, voxels should be in between 0.25 mm and 1.25 mm; however, optimal size will vary depending on the pathology of interest. For example, reconstructing an orbital floor will require thinner voxels than cardiac tissue. Therefore, physicians should keep in mind the anatomy of interest while establishing image-acquisition protocols [5].

### 2.2. Image Segmentation and Postprocessing

**Figure 2:**
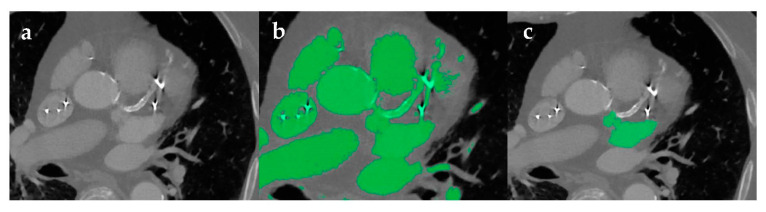
Image segmentation process with artifacts. (**a**) Axial contrast enhanced CT of the heart demonstrates bright artifacts as a result of a metal implant and arterial calcification. (**b**) These artifacts diminish the efficacy of automatic segmentation via thresholding. (**c**) Manual segmentation was used to isolate the area of interest.

Following their acquisition, medical images are stored as Digital Imaging and Communications in Medicine (DICOM) files. In order to generate a 3D file compatible with printers, DICOM images can be manipulated using segmentation and postprocessing software packages such as MeVisLab (Mevis Medical Solutions, Bremen, Germany) or Mimics (Materialise, Leuven, Belgium). These software allow for the segmentation and isolation of select anatomical structures using automatic, semi-automatic, and manual techniques [2,8], with the pathology of interest dictating which techniques are used. Methods like thresholding may be sufficient for isolating skeletal features on a CT scan due to the high attenuation characteristics seen in bony structures. However, further manipulation is often necessary to segment soft tissues. Holes can form in the final model due to the heterogeneous, low signal intensity of various pathologies and imprecise thresholding. Streak artifacts and beam hardening from air, metal implants, embolization coils, or high-density blood are challenges for 3D modeling. In instances where these artifacts cannot be fixed during image acquisition, additional segmentation techniques must be implemented (Figure 2). For example, region growing is a useful tool for automated segmentation, allowing the user to confirm whether selected voxels are truly categorized within the segmented “part”. This reduces the amount of manual manipulation or “sculpting” required in the later stages of segmentation [5].

Most software packages automatically create a 3D STL file after segmentation of the individual DICOM images is complete. This file is generated by complex algorithms like interpolation, which combines the segmented regions of interest into a 3D model. With STL files, users can define the number of triangles that comprise each surface. Similar to voxel size, it is important to take into account the anatomy of the model. An inadequate number of triangles or “facets” will result in an anatomically inaccurate model. However, depending on the type of model, surpassing a threshold number of triangles will have no benefit to accuracy, increase postprocessing time, and may result in a rough, jagged surface. Highly vascularized models will have a higher threshold maximum of facets than simpler morphology [5].

**Figure 3:**
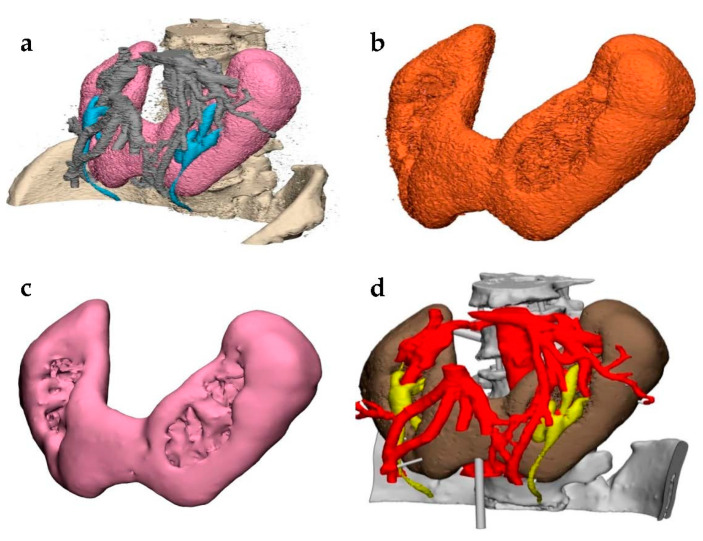
STL file postprocessing in a model demonstrating renal fusion, i.e., horseshoe kidney. (**a**) The segmented medical images were converted into a STL file and imported into a postprocessing software. (**b**) The desired shell was selected and inverted to highlight and delete floating artifacts around the model. Techniques such as “wrapping” were performed to allow for an initial smoothing. Local smoothing was used to further refine the isolated part into (**c**) a final shell. (**d**) This process was repeated for each part of the model until it was completed and ready for printing.

Despite being compatible with 3D printers, the generated 3D STL file often requires alterations before it is ready to be printed (Figure 3) [9]. Mistakes in the segmentation process can lead to defects in the 3D model, such as holes between facets or inverted normals, where the inside and outside surface of the model is flipped. A common cause of errors in STL files is due to their definition of a surface, which requires that any surface must enclose a space. For this reason, open regions of interest cannot be rendered in this format and users must manually edit the STL file using computer-aided design (CAD) software, in order to “close” the area of interest and thus make it manifold [5]. Furthermore, the creation of additional components may be necessary to stabilize the 3D model once printed. These parts can be created using CAD software [9]. Such techniques may require expertise in order to correctly implement without altering the anatomical accuracy of the model itself [5]. The RSNA 3D Printing SIG suggests that physicians carefully document alterations made during postprocessing, categorizing these alterations into clinically relevant and clinically irrelevant changes in order to ensure full clarity during the 3D-modeling process [7].

### 2.3. 3D Printing

3D printers use information from the STL file to divide the model into successive cross-sections, deposit selected regions of interest within each layer, and fuse them together into a physical structure [5]. The RSNA 3D Printing SIG recommends that each anatomy be modeled in at least three separate layers. For example, if the smallest pathology physicians are interested in is a lesion 3 mm large, layer thickness should be 1 mm at the most (although preferably much less). In addition, optimal layer thickness will vary depending on the model’s required accuracy [7].

There are several types of 3D-printing technologies, the majority of which are solid, liquid, or powder-based [10]. When selecting which 3D-printing technique to use for an anatomical model, it is important to consider factors such as availability, printing time, cost, and purpose of the model. Here, we discuss the five most accepted and commonly used technologies: stereolithography, binder jetting, material jetting, material extrusion, and powder-bed fusion. Other 3D-printing techniques, such as sheet lamination and directed energy deposition, exist. However, they are uncommonly used in the medical field.

#### 2.3.1. Stereolithography

Stereolithography (SLA), also referred to as digital light processing or vat photopolymerization, is one of the oldest forms of 3D printing and is often used in the creation of anatomical models for preoperative planning [5,9]. Layers of photosensitive liquid resin are exposed one after another to a high-intensity light source, each layer solidifying over the previous until the entire model is created. Once solidified, the model is left in an ultraviolet chamber for a final curing. SLA is most commonly used to model bony structures due to the clear to opaque white appearance of its photopolymer materials. This appearance is advantageous for visualizing internal anatomies (i.e., teeth, nerves, tumors), and allows for selective darkening by overexposing the material to light. However, extra steps such as sanding, manual removal of support structures, applying an ultraviolet sealant, and rinsing in a cleaning solution can increase the cost and time to produce models using this method [5]. In addition, the necessity for photosensitive material results in a limited range of materials for printing [9].

#### 2.3.2. Binder Jetting

Binder jetting (BJG) works by spraying a liquid binding agent onto a bed of powdered material (most notably metal alloys or ceramics) [11]. After one layer has hardened, a new bed of powder is deposited and the process begins again, thus creating layer upon layer until the final model is complete. BJG has multiple advantages. During the 3D-printing process there is no need for added supports due to the bed of powder, which acts as a support itself. In addition, this process is often used to create colored models given its range of inexpensive, colored materials. “Infiltration” of BJG models using an elastomer can yield deformable constructs [5]. Nevertheless, the current unavailability of translucent materials makes visualization of internal structures difficult. The models are often less stable and durable than those created by other technologies. Additionally, BJG requires extensive postprocessing (sintering, de-powdering, infiltration etc.), greatly adding to the time it takes to print [11].

#### 2.3.3. Material Jetting

Material jetting (MJ), also referred to as polyjet or multijet printing [12], is similar to BJG. This technique uses jets to spray a liquid, photosensitive polymer onto a tray, and then exposes the layer to ultraviolet light for curing. Each layer is cured successively on top of one another, until the model is complete. Supports can be created out of a gel-like substance and are easily removed with a soap solution after printing is complete. Additionally, the materials used in MJ are versatile and are available in an array of colors, allowing for more customized and detailed anatomical models. Materials can be mixed to alter their physical properties, which has been noted as particularly useful for resembling skin and in orthodontic applications [5]. However, it is important to note the expiration dates of these photosensitive polymers, as most printers do not allow their use following expiration [5].

#### 2.3.4. Material Extrusion

Material extrusion methods such as fused deposition modeling (FDM) are the most commonly used form of 3D printing, due to their low cost and ease of use [13]. Material extrusion works by extruding heated materials (plastic, metal, etc.) layer after layer onto a tray. As the material cools, it fuses and hardens with the rest of the model. This process is used less in the medical field in comparison to other industries due to the low quality and resolution of its models. However, this technique may be useful in cases where a quick, inexpensive, less-detailed model is necessary [5].

#### 2.3.5. Powder-Bed Fusion

Powder-bed fusion includes popular technologies such as selective laser sintering (SLS) and selective laser melting (SLM). These techniques utilize high-powered lasers to melt particles of material together into a uniform layer. After this, new powder is deposited on top of that layer and the fusion process repeats, joining individual particles within successive layers. Powder-bed fusion is commonly used to create medical implants, due to the durability of the models. In addition, it allows for the creation of more complex morphologies (for example lattices), which other processes may have difficulty printing. Postprocessing depends on the materials used to create the 3D model. For instance, metals may require extensive finishing using industrial-level milling technology [5].

## 3. Clinical Applications in Preoperative Planning

As a result of recent innovations, 3D printing has already shown widespread success in surgical planning. A systematic review found that almost 40% of publications discussing the uses of 3D printing in medical settings included 3D modeling and its application in preoperative planning. These studies demonstrated the correlation of 3D models to patient-specific anatomical structures, decreased operation time, decreased exposure to ionizing radiation, and benefits in patient outcome [3], most notably in the practice of cardiovascular surgery, neurosurgery, craniomaxillofacial surgery, orthopedic surgery, and interventional radiology.

### 3.1. Cardiovascular Surgery

MRI, CT, and echocardiography are the main modalities of cardiac imaging. Although they provide high resolution, these imaging methods fall short when faced with the diverse morphologies of cardiac disorders. Patients with a history of cardiac surgery are at high risk for unanticipated surgical outcomes due to the lack of information standard imaging provides [2]. Thus, patient-specific models can greatly aid a physician’s preparation prior to surgery. 3D models have already shown promising results, particularly in the surgical planning of patients with congenital heart defects like atrial and ventricular septal defects [14,15,16,17,18,19]. CT angiography-based 3D-printed models are used to plan the transcatheter closure of pathologies such as muscular ventricular septal defects, allowing clinicians to accurately estimate catheter size prior to the operation [20]. Ryan et al. used CT imaging and a 3D-printed model alone to guide an intervention in a neonatal presenting tetralogy of Fallot (TOF) with pulmonary valve atresia and multiple aortopulmonary collateral arteries. This severe variation of TOF usually requires significant invasive examination of patient morphology prior to surgery; however, 3D printing has proved to be a successful alternative to traditional TOF diagnostic protocols [21].

**Figure 4:**
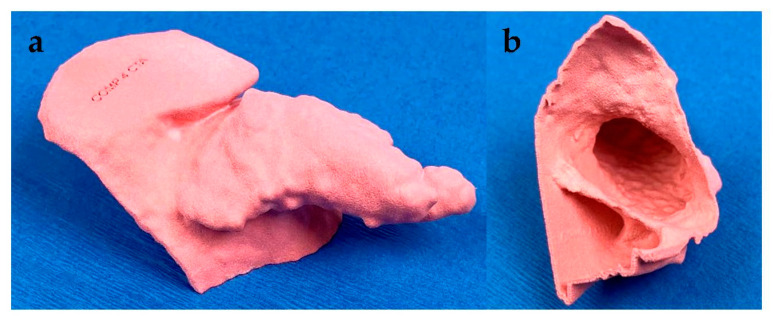
Left atrial appendage (LAA) model. Segmentation and 3D printing allow rapid and accurate sizing of optimal LAA occlusion devices. (**a**) Accurate external and (**b**) internal view of the 3D printed LAA model assisted with sizing and placement of the occlusion device.

Not only do 3D models provide physicians with accurate visualization of various cardiac lesions, but they also improve implant positioning and sizing. Several studies have demonstrated the potential benefits of using 3D models to predict implant size and placement during left atrial appendage (LAA) occlusion procedures (Figure 4) [22,23,24,25]. Complex operations like transcatheter valve replacements can also be optimized using 3D printing [26,27]. Transcatheter aortic valve replacement (TAVR) requires extensive data on aortic morphology; misplacement of the prosthetic valve may result in paravalvular leaks, coronary artery obstruction, and other adverse outcomes. Physical models allow for benchtop testing of prosthetic valve placement and can successfully predict the presence, location, and etiology of complications [28]. Using 3D-printed models to rehearse long-term prosthetic valve implantation in the presence of calcification, Fujita et al. elucidated the causal relationship between asymmetric calcification of the left coronary cusp and a need for permanent pacemaker implantation post-TAVR. Models demonstrated each prosthetic valve’s gradual movement to the right, eventually leading to interference with the cardiac conduction system [29].

As the use of 3D cardiac models becomes more prevalent, new innovations improve upon existing 3D-printing techniques. Advancements in 3D echocardiography have led to higher resolution 3D models that can demonstrate minute features of heart disorders such as valvular leaks [2]. Furthermore, multi-material 3D printing may allow for more realistic surgical simulation; one study used a combination of different elastomeric materials to generate 3D-printed mitral valves which closely resembled the physical properties of a true valve [30].

### 3.2. Neurosurgery

The goal of 3D printing in neurosurgery is to determine the optimal approach to surgical procedures [2]. Doing so requires detailed information on surrounding anatomical structures. MRIs provide a wealth of data, thanks to their high contrast and spatial resolution of the brain and spinal cord. The creation of 3D imaging modalities in combination with virtual reality simulations have further benefited preoperative planning, as 2D displays of such renderings do not accurately resemble 3D depth and lack tactile experience, which could later aid a physician in the surgical suite [2,31]. The multicolored models of 3D printing techniques, such as binder jetting, illuminate small anatomical structures that medical imaging may not adequately resolve, and can be used to determine the course of intervention [5].

Such models have enhanced the preoperative planning of neurovascular procedures in multiple studies, providing detailed representation of patient-specific vasculature [32,33,34,35]. Intervention in patients with intracranial aneurysms are particularly complex procedures, and in cases where embolization is not possible, surgeons use microsurgical clipping to restrict blood flow. A retrospective study used CT angiography to generate 3D-printed models and determine their efficacy in simulating the reality of the surgical suite. The models demonstrated accurate aneurysm clip sizing in comparison to the actual clips used during surgery and proved beneficial towards replicating patient morphology [36]. In addition, simulation of cerebrovascular intervention for educational and clinical purposes is possible thanks to the advent of 3D-printed vascular networks. Mashiko et al. successfully used such networks to simulate aneurysm clipping and provide trainees with practical, hands-on experience [37].

The planning of functional neural interventions, such as subdural electrode implantation in patients with epilepsy, does not usually involve patient-specific positioning to optimize patient evaluation. However, high-precision 3D-printed models of patients’ cerebrums allowed physicians to customize electrode arrays based on an individual’s gyral and sulcal features. Morris and colleagues were also able to vary electrode density per array in order to cluster electrodes around anatomies of interest, a useful innovation for both clinical and research purposes. For instance, to focus on the physiology of the motor cortex, each array contained a greater density of electrodes around the motor bank of a patient’s central sulcus [38].

### 3.3. Craniomaxillofacial Surgery

Similar to neurosurgery, craniomaxillofacial surgery often relies on preoperative surgical simulation to determine the best route of intervention, however virtual simulations have several limitations, leading to inaccurate assessment of skeletal deformations, development, and positioning [2]. These errors prove especially difficult during the preoperative planning of osteotomies, where accurate anatomical information is critical. 3D-printed models were used to successfully simulate osteotomies prior to the operation and shape titanium implants, which are difficult to adjust intraoperatively [39]. Otologic treatments such as tympanoplasty also pose a challenge during preoperative planning due to the ear’s complicated morphology. Numerous studies report the creation of 3D-printed models to replicate ear deformation, testing these models as a more realistic basis for surgical simulation and training [40,41,42,43,44]. Hochman et al. used 3D printing to model temporal bones and compared the efficacy of their printed models for surgical simulation with virtual models; residents found the printed model to be a more effective tool for recreating surgical settings [41].

### 3.4. Orthopedic Surgery

Orthopedic surgery commonly uses medical 3D printing. In a systematic review, orthopedic surgeries accounted for almost 25% of publications reporting on the surgical contributions of 3D printing [3]. Surgical treatment of fractures is an area of particular interest, with models demonstrating improvements in the duration of the operation and minimized blood loss [45,46,47]. One study demonstrated the benefit of 3D-printed models in optimizing the surgical approach for distal tibial fractures. They reported 74% of inexperienced surgeons altered their initial plate selection for distal tibial fracture surgery upon receiving a 3D-printed model of their patient’s anatomy; 9% of experienced surgeons switched plate selection [48]. Orthopedic applications of 3D printing also include the creation of oncologic models for tumor resection; these can facilitate tumor resection and accurately replicate the lesion of interest along with surrounding anatomy. Furthermore, 3D-printed models could aid the design of allografts replacing cancerous tissue [5].

### 3.5. Interventional Radiology

**Figure 5:**
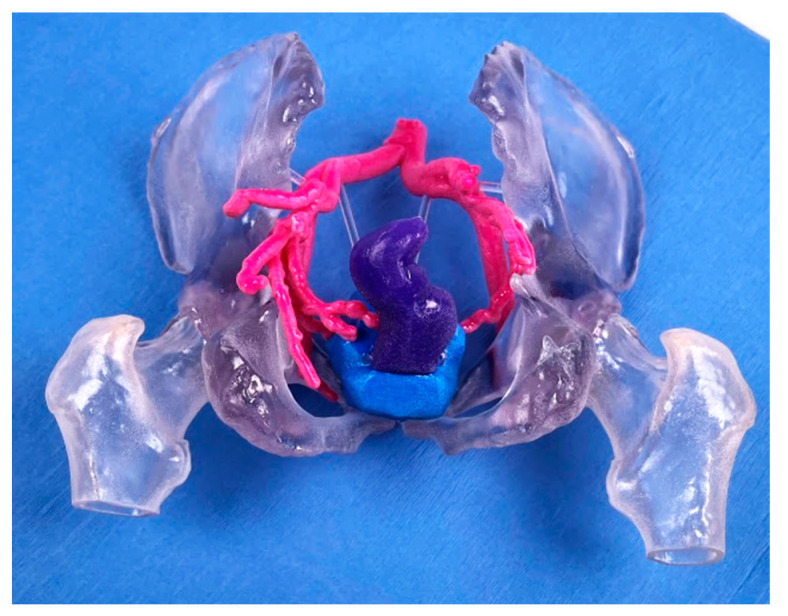
3D-printed model for prostate artery embolization. The model demonstrates the colon (purple), prostate (blue), and surrounding arterial vessels (pink) allowing physicians to optimize their embolization approach prior to the procedure.

The use of accurate models to guide physicians is critical in interventional radiology (IR). Renderings in three dimensions lack the spatial resolution necessary to optimize IR procedures such as embolization, which require detailed understanding of patient anatomy [49]. In addition to previously mentioned IR applications in cardiovascular surgery, Giannopoulos et al. 3D-printed a patient’s ascending aorta and aortic arch in order to simulate an endovascular repair [50]. 3D printing was also used to model splenic artery aneurysms and test a variety of catheterized approaches. By determining the best approach prior to the operation, physicians were able to decrease operation duration and patient exposure to radiation. In addition, some approaches that were attempted, such as coil embolization, may have led to arterial injury if tested in the surgical suite [51]. A 3D-printed model to guide prostate artery embolization is shown in Figure 5; the model clearly depicted a branch of the prostate artery perfusing the rectum. These models not only allow for planning of the procedure, but also act as a visual tool to describe pertinent pathology to the patient and the approach that will be taken to treat it.

### 3.6. Other Interventions

The success of whole organ transplant requires an understanding of patient anatomy prior to surgery; a recipient’s surrounding anatomy should be compatible with the donor’s to ensure a good surgical outcome. Zein et al. tested the efficacy of 3D-printed livers as a potential method for surgical planning in liver transplantation. These models proved to be accurate replications of patient-specific biliary structures and vasculature [52]. Chandak et al. 3D-printed pediatric kidneys to assess transplant feasibility in three patients presenting stage 5 chronic renal failure. While traditional imaging was not able to determine each patient’s candidacy for kidney transplants, the printed models successfully overcame this challenge. In addition, the models were used to identify the optimal recipient vessel for anastomosis [53].

Silberstein et al. used 3D printing in renal tumor resection. Patient kidneys and their associated tumors were modeled to guide physicians in their preoperative planning. These models also facilitated patient education, providing a visual aid when discussing the course of intervention [54].

## 4. Future Perspectives

There are numerous applications of 3D printing in preoperative planning. As this field continues to grow, it is critical that physicians familiarize themselves with the fundamentals. Although 3D-printed models are already outperforming conventional methodologies for surgical planning and rehearsal, challenges still remain. Currently, 3D printing is limited in its ability to mimic various tissues [2]. Although material mixing has made strides towards customizing the mechanical properties of 3D models, mimicking the physical characteristics of in vivo tissue remains difficult. Bioprinting may be able to address some of these issues, allowing physicians to create models with physiological heterogeneity, while still controlling material properties on macro and micro levels [55]. Another innovative solution is 4D printing, combining 3D printing techniques with smart materials that change properties depending on mechanical, thermal, chemical, and electrical stimuli [56]. However, the use of bioprinting and 4D printing in clinical settings is rare and still faces many of the logistical obstacles that 3D printing faces. For example, Yan and colleagues have pointed to the lack of standardized metrics for evaluating 3D-printed materials as an obstacle for diversifying 3D printing’s usage. They encourage further research to focus on this [57]. In addition, these technologies are still a costly, albeit more accurate, alternative to current surgical planning [4], making hospitals hesitant to adopt it on a large scale. The overall cost of 3D printing continues to decline as technologies become more available. However, the high expense of starting an in-center 3D printing laboratory and its slow economic gain will limit 3D printing’s utility in the medical field [5].

The traditional workflow of 3D printing is another barrier towards full implementation. Current 3D-printing technologies and software require extensive expertise in computer-based design to use effectively. This may require physicians to individually gain in-depth knowledge about the 3D-printing process until future training programs and infrastructures address 3D printing’s need for technical and medical expertise [5]. Some workflows task engineers with the actual creation of 3D models, while physicians confirm anatomical accuracy during segmentation and again after the model has been finalized. However, even these optimized workflows require successful collaboration between engineers and physicians, and a mutual understanding of the medical and technical aspects of 3D printing is optimal until the need for more navigable software is met [10].
